# A hot hole-programmed and low-temperature-formed SONOS flash memory

**DOI:** 10.1186/1556-276X-8-340

**Published:** 2013-07-31

**Authors:** Yuan-Ming Chang, Wen-Luh Yang, Sheng-Hsien Liu, Yu-Ping Hsiao, Jia-Yo Wu, Chi-Chang Wu

**Affiliations:** 1 Department of Electronic Engineering, Feng Chia University, Taichung 407, Taiwan; 2 Ph.D. Program in Electrical and Communications Engineering, Feng Chia University, Taichung 407, Taiwan; 3 Department of Dentistry, Taipei Medical University Hospital, Taipei 110, Taiwan; 4 School of Dentistry, College of Oral Medicine, Taipei Medical University, Taipei 110, Taiwan; 5 Graduate Institute of Biomedical Materials and Tissue Engineering, College of Oral Medicine, Taipei Medical University, Taipei 110, Taiwan

**Keywords:** Sol–gel, Hole trapping, Flash memory

## Abstract

In this study, a high-performance Ti_*x*_Zr_*y*_Si_*z*_O flash memory is demonstrated using a sol–gel spin-coating method and formed under a low annealing temperature. The high-efficiency charge storage layer is formed by depositing a well-mixed solution of titanium tetrachloride, silicon tetrachloride, and zirconium tetrachloride, followed by 60 s of annealing at 600°C. The flash memory exhibits a noteworthy hot hole trapping characteristic and excellent electrical properties regarding memory window, program/erase speeds, and charge retention. At only 6-V operation, the program/erase speeds can be as fast as 120:5.2 μs with a 2-V shift, and the memory window can be up to 8 V. The retention times are extrapolated to 10^6^ s with only 5% (at 85°C) and 10% (at 125°C) charge loss. The barrier height of the Ti_*x*_Zr_*y*_Si_*z*_O film is demonstrated to be 1.15 eV for hole trapping, through the extraction of the Poole-Frenkel current. The excellent performance of the memory is attributed to high trapping sites of the low-temperature-annealed, high-*κ* sol–gel film.

## Background

Silicon-oxide-nitride-oxide-silicon (SONOS)-type memory is widely used for nonvolatile memory [1]. Compared to conventional floating-gate memory, SONOS-type memory has the advantage of high date retention, high endurance, and fast program/erase (P/E) speed [2]. However, the primary drawback of this memory type is that a higher voltage (typically >10 V) is required to inject carriers into the charge trapping layer, which results in excessive power consumption and leakage current. A device with low operation voltage is necessary for the development of high-performance memory [3].

Recently, high-*κ* materials have been considered as an effective charge storage material to achieve a faster program speed and improved charge retention [4,5]. Numerous technologies have been developed for the preparation of various high-*κ* films, including the sol–gel method, atomic layer deposition, physical vapor deposition, and chemical vapor deposition [6-9]. Among them, the sol–gel method is an appealing technique. Using this method, the high-*κ* film can be easily synthesized by mixing many types of materials in a solvent, followed by a post-anneal process after spin-coating on a substrate [10]. The advantages of the sol–gel method include simplicity, low cost, good uniformity, and compatibility with the current production lines of semiconductor plants [11]. However, performing high-temperature post-annealing to obtain a satisfying high-*κ* film was unavoidable in previous studies [6,10-13]. The high-temperature post-annealing, which is typically above 900°C, hinders the wide application of the sol–gel method, such as in thin-film transistors or flexible devices.

In this study, a high-quality Ti_*x*_Zr_*y*_Si_*z*_O film was synthesized using the sol–gel method and low-temperature post-anneal. The sol–gel-derived Ti_*x*_Zr_*y*_Si_*z*_O film was applied as the charge storage layer of the SONOS-type flash memory. Identical to the high-temperature sample, the low-temperature post-annealed memory shows a noteworthy hot hole trapping characteristic and exhibits a lower operation voltage, faster P/E speed, and better data retention than previously demonstrated.

## Methods

The fabrication of sol–gel-derived memory was started with a local oxidation of silicon isolation process on a p-type (100), 6-in. Si substrate. A 4-nm tunneling oxide was thermally grown at 925°C in a furnace. A sol–gel solution containing zirconium tetrachloride (ZrCl_4_), silicon tetrachloride (SiCl_4_), and titanium tetrachloride (TiCl_4_) was then spin-coated onto the substrate at 3,000 rpm for 60 s at ambient temperature. The sol–gel solution used ethanol as the solvent, and the molar ratio of the mixture for ZrCl_4_/SiCl_4_/TiCl_4_/ethanol was 1:1:1:1,000.

**Figure 1:**
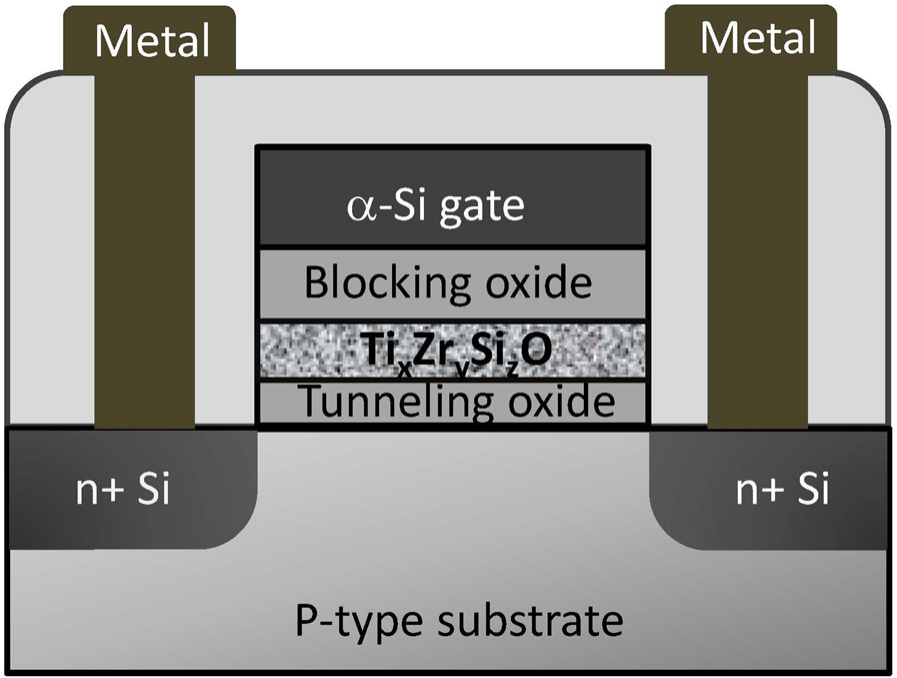
**Schematic structure of the Ti**_***x***_**Zr**_***y***_**Si**_***z ***_**O flash memory.** The Ti_*x*_Zr_*y*_Si_*z *_O thin film was used here as the charge trapping layer.

After the sol–gel film was coated, a rapid thermal annealing (RTA) process was conducted at 600°C for 60 s in an oxygen ambience. During the RTA process, a compound layer of metal-oxide-silicate containing titanium and zirconium was formed. A 10-nm blocking oxide film and 200-nm amorphous Si film were then deposited subsequently. The blocking oxide was grown by plasma-enhanced chemical vapor deposition, using silane (SiH_4_) and nitrous oxide (N_2_O) as the precursors to form a 10-nm SiO_2_. The 200-nm amorphous Si film, used as the gate electrode, was deposited in the same system using the SiH_4_ precursor. After gate patterning, As^+^ ions were implanted at 20 keV with a dosage of 5E15 cm^−2^ and annealed at 600°C for 24 h to define the source and drain. Finally, a 500-nm tetraethyl orthosilicate oxide was formed as the passivation layer, and the subsequent processes were used to fabricate the memory. The schematic structure of the Ti_*x*_Zr_*y*_Si_*z*_O flash memory is shown in Figure 1. The channel width and length of the memory were 10 and 0.35 μm, respectively.

## Results and discussion

**Figure 2:**
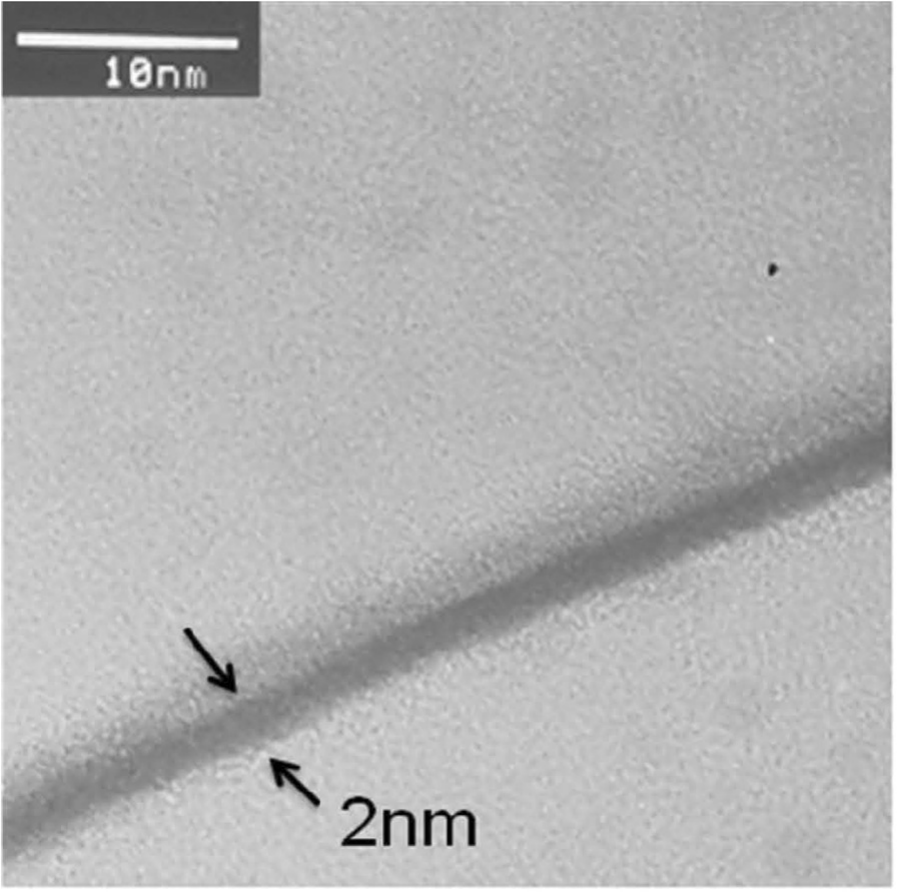
**Cross-sectional TEM image of the sol–gel-derived Ti**_***x***_**Zr**_***y***_**Si**_***z***_**O film.** The thickness of the Ti_*x*_Zr_*y*_Si_*z *_O film is calculated to be 2 nm after 600°C annealing.

**Figure 3:**
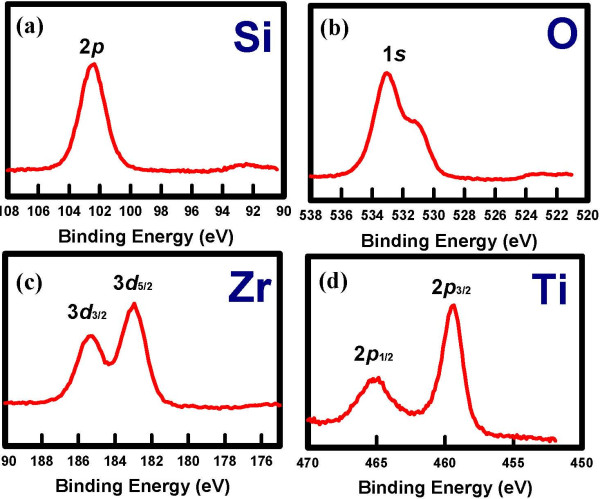
**XPS spectra of the sol–gel-derived Ti**_***x***_**Zr**_***y***_**Si**_***z ***_**O film. (a)** Si 2*p*, **(b)** O 1*s*, **(c)** Zr 3*d*, and **(d)** Ti 2*p* spectra.

Figure 2 shows the cross-sectional transmission electron microscopy (TEM) image of the sol–gel-derived Ti_*x*_Zr_*y*_Si_*z*_O film annealed at 600°C. A continuous and smooth film of 2 nm in thickness was observed, suggesting that no obvious film morphology occurred in the sample annealed at 600°C. The composition of the sol–gel-derived Ti_*x*_Zr_*y*_Si_*z*_O film was analyzed by X-ray photoelectron spectroscopy (XPS), and the Si 2*p*, O 1*s*, Zr 3*d*, and Ti 2*p* spectra of the Ti_*x*_Zr_*y*_Si_*z*_O film are shown in Figure 3a,b,c,d, respectively. The peaks in the figures indicate the component formation of the Ti_*x*_Zr_*y*_Si_*z*_O film.

**Figure 4:**
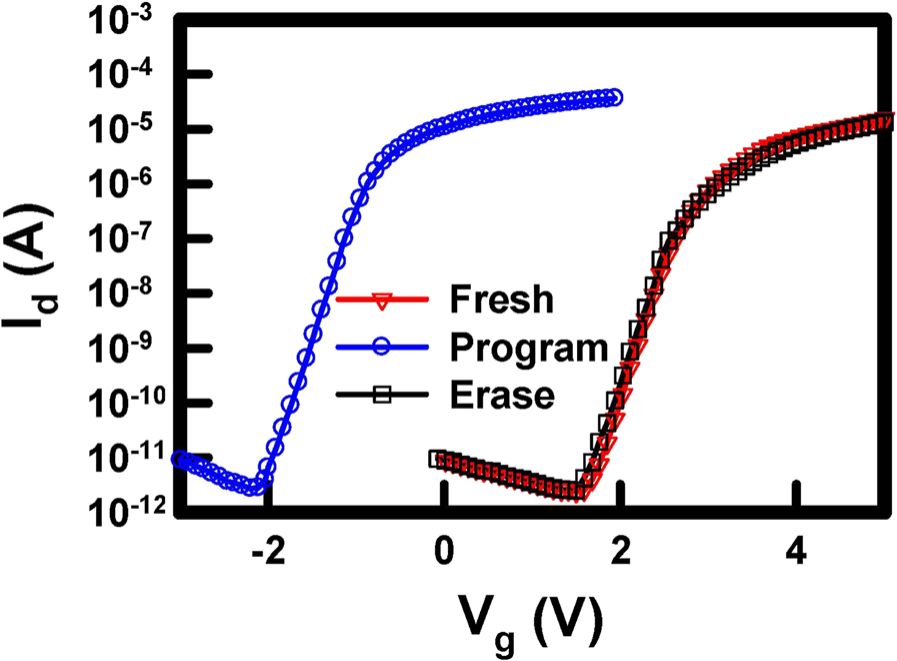
***I***_**d**_**-*V***_**g**_**characteristics of the sol–gel-derived Ti**_***x***_**Zr**_***y***_**Si**_***z ***_**O memory at fresh, program, and erase states.** The memory window is *ca.* 3.7 V.

Figure 4 shows the *I*_d_-*V*_g_ curves of the Ti_*x*_Zr_*y*_Si_*z*_O memory in fresh, program, and erase states. The measured condition for the program operation was *V*_g_ = −8 V, *V*_d_ = 8 V, and 1 ms, and that for the erase operation was *V*_g_ = 8 V, *V*_d_ = 8 V, and 1 ms. The characteristic curve shows a 3.7-V leftward shift after the program operation and then a shift back to the original, fresh state after the erase operation. Instead of applying a positive gate bias for programming previous cases, a negative gate bias was used to program the Ti_*x*_Zr_*y*_Si_*z*_O memory. That is, a band-band hot hole (BBHH) was used to program, whereas a channel hot electron (CHE) was used to erase this memory. Programming was also attempted by injecting the electrons into the charge trapping layer, according to the method most previous studies reported, by applying a positive voltage to both gate and drain electrodes. However, only a minimal shift of the curve was observed.

**Figure 5:**
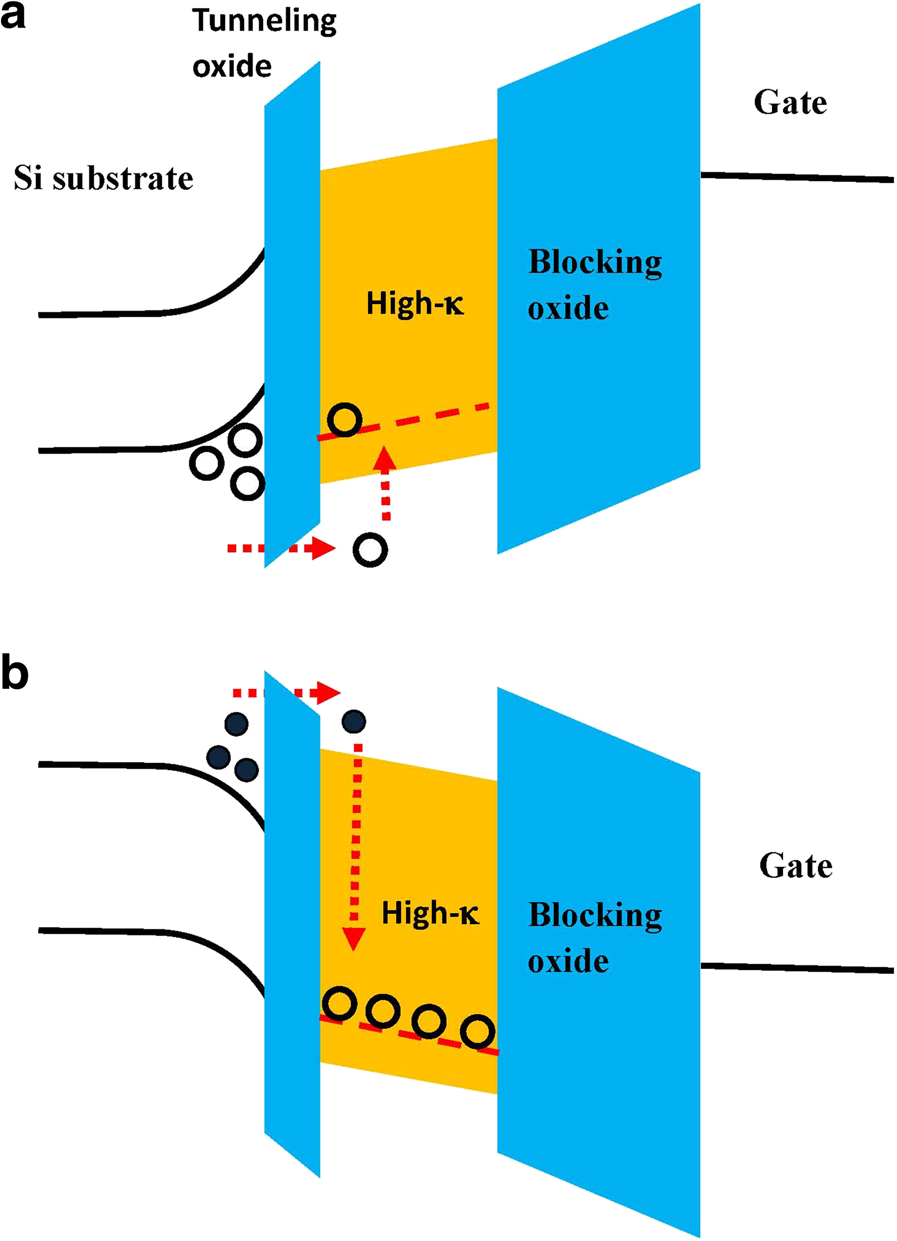
**Band diagrams of the Ti**_***x***_**Zr**_***y***_**Si**_***z ***_**O memory in the (a) program and (b) erase operations.**

Based on the *I*_d_-*V*_g_ measurement results, band diagrams of the Ti_*x*_Zr_*y*_Si_*z*_O memory in the program and erase operations are illustrated in Figure 5a,b, respectively. For the program operation, a BBHH was used; therefore, hot holes were injected from the silicon substrate and captured by the hole traps in the charge trapping layer, as shown in Figure 5a. In the erase operation, positive gate and drain voltages were applied. Channel hot electrons were injected and then recombined with the holes in the trap site, as shown in Figure 5b.

To demonstrate the thermal emission of carriers in the trap of the Ti_*x*_Zr_*y*_Si_*z*_O memory, the Poole-Frenkel current was measured. The Poole-Frenkel current explains the hot hole trapping effect of the memory [14,15]. The expression for current density according to the Poole-Frenkel emission can be written as [16]:

$$J_{\mathrm{FP}}=aE_{\mathrm{ox}}\exp\left[\left({\left(bE_{\mathrm{ox}}\right)}^{1/2}-\varphi_{t}\right)q/k_{b}T\right],$$

where *K*_b_, *T*, *a*, *b*, and *φ*_*t*_ are the Boltzmann constant, the measurement temperature, a constant that depends on the trap density, a constant that depends on the electric permittivity, and the depth of the trap potential well, respectively.

**Figure 6:**
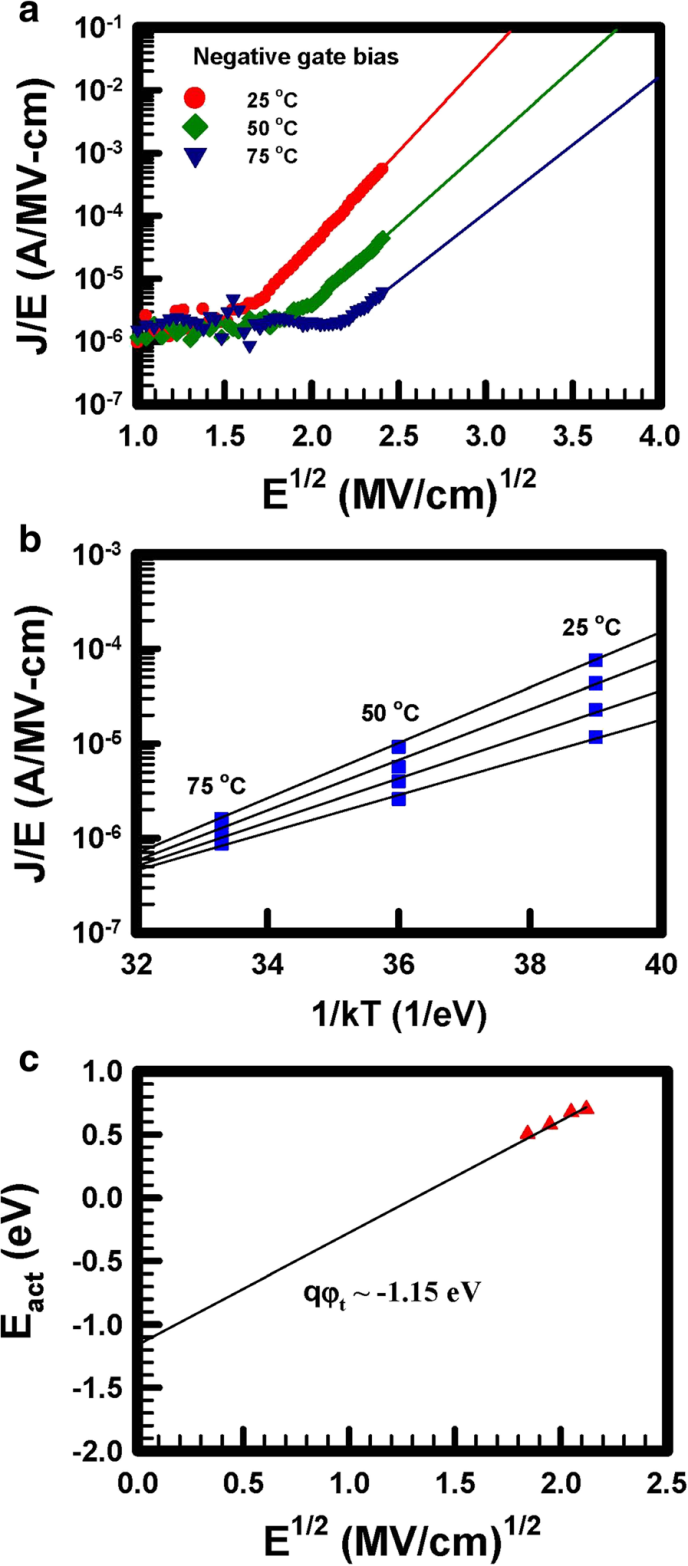
**Poole-Frenkel current of the Ti**_***x***_**Zr**_***y***_**Si**_***z***_**O memory under negative gate bias. (a)** Poole-Frenkel plot of the Ti_*x*_Zr_*y*_Si_*z *_O memory at different measuring temperatures. **(b)** Arrhenius plot of the memory at different values of electric field. **(c)** Graphical determination of the trap depth from the dependence of activation energy on the square root of electric field.

If hot hole trapping is the dominant mechanism for programming the Ti_*x*_Zr_*y*_Si_*z*_O memory, the extracted current should follow the Poole-Frenkel emission, that is, a linear slope for the plot of current density (*J*/E) versus the square root of the applied electrical field. Therefore, a negative bias from 0 to −20 V was applied to the gate electrode with a constant 4-V drain bias at measurement to simulate the hot hole program of the memory. Figure 6a shows the plot of current density versus the square root of the applied electrical field under various measuring temperatures at hot hole program operation. Linear regions of the plot imply that the current of Ti_*x*_Zr_*y*_Si_*z*_O memory follows the Poole-Frenkel emission. Figure 6b shows an Arrhenius plot of the memory extracted from Figure 6a. The linear dependence of the current densities versus temperatures implies that the charges exhibit a thermally activated behavior, which is consistent with the Poole-Frenkel emission. The barrier height of the Ti_*x*_Zr_*y*_Si_*z*_O film to silicon oxide can be extracted as approximately 1.15 eV for hole trapping, using the Poole-Frenkel current, which is shown in Figure 6c.

**Figure 7:**
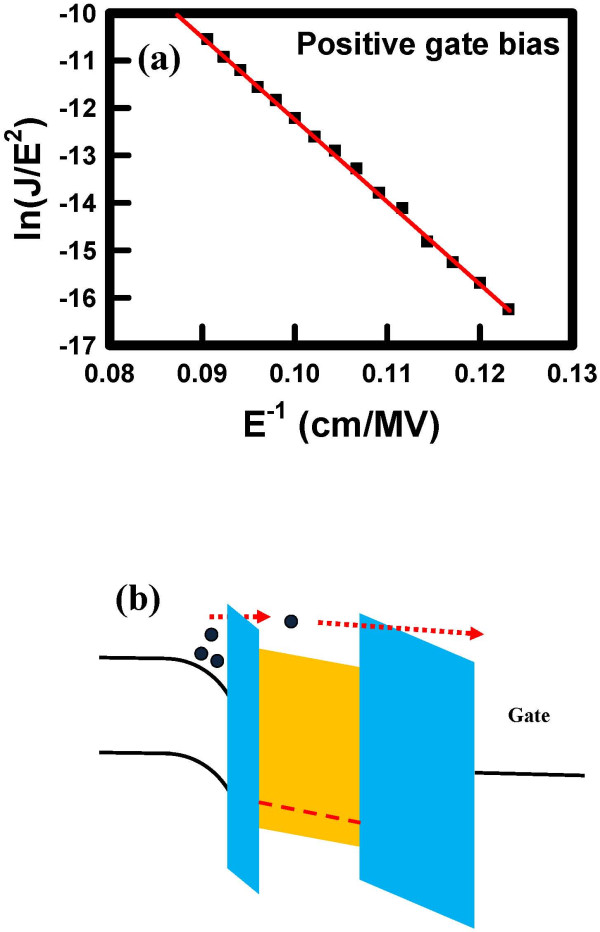
**Fowler-Nordheim plot (a) and band diagram (b) of the Ti**_***x***_**Zr**_***y***_**Si**_***z ***_**O memory under positive gate bias.** The linear dependence indicates that FN tunneling is dominant under positive bias.

In addition to hot hole trapping, the Poole-Frenkel current of the hot electron program was also measured by applying a positive gate voltage. However, the result showed a nonlinear curve. Conversely, the measured result showed a linear dependence of current density, divided by the electric field squared, versus the reciprocal electric field (Figure 7a), which is represented by Fowler-Nordheim tunneling. This result may indicate that the energy band of the Ti_*x*_Zr_*y*_Si_*z*_O film exhibits shallow trap potential well that could not preserve electrons when applying a positive gate voltage. Therefore, electrons were injected into the charge trapping layer and then went through the blocking oxide to the gate electrode. The band diagram of the Fowler-Nordheim (FN) operation is illustrated in Figure 7b. The expression of Fowler-Nordheim tunneling on an electric field can be given by [17]:

$$J_{\mathrm{FN}}=cE_{\mathrm{ox}}{}^{2}\exp\left(-d/E_{\mathrm{ox}}\right),$$

where *c* represents a constant that depends on the energy barrier height and *d* is a constant that depends on the electric effective mass for tunneling.

**Figure 8:**
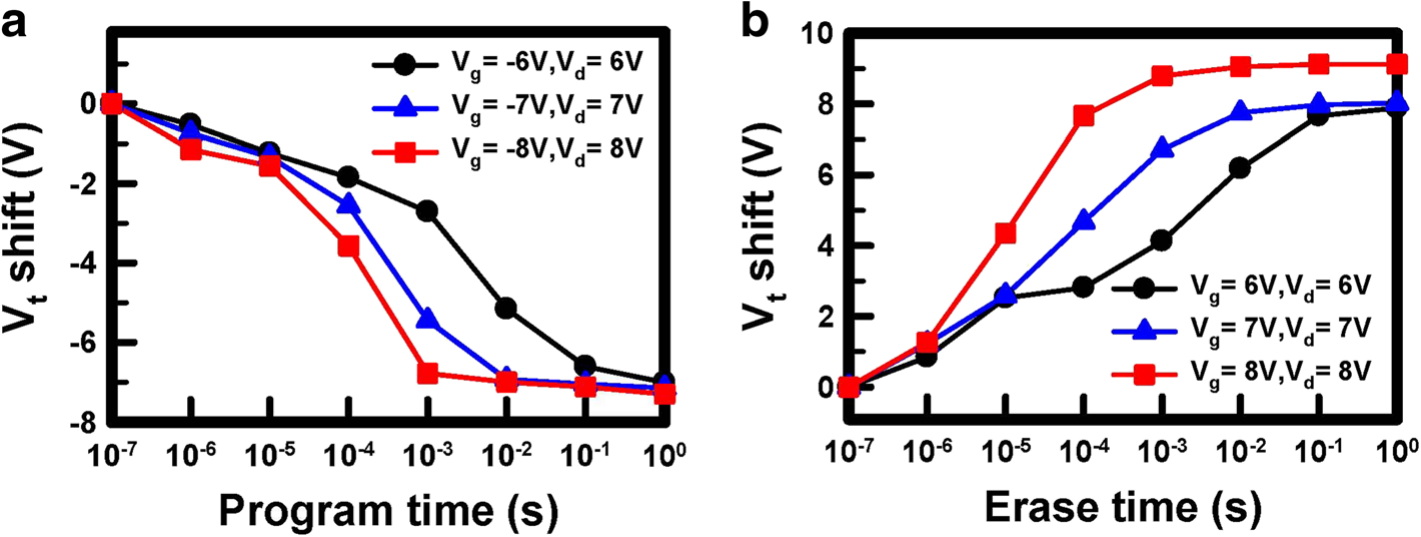
**Program (a) and erase (b) speeds of the Ti**_***x***_**Zr**_***y***_**Si**_***z ***_**O memory under various operation conditions.** The program and erase speeds for a 2-V voltage shift are 16 and 1.7 μs, respectively.

Figure 8a,b shows the program and erase speeds, respectively, of the Ti_*x*_Zr_*y*_Si_*z*_O memory under various operation conditions. Because the memory exhibited the hot hole trapping property, BBHH was applied to programming and CHE was applied to erasing.

As shown in Figure 8a, the threshold voltage (*V*_t_) shift increased with increasing operation voltage; therefore, more ‘hot’ holes were generated and injected into the charge storage layer. The maximum memory window can be as large as 8 V. The program speed is 16 μs with a −2-V *V*_t_ shift for the program conditions of *V*_g_ = −8 V and *V*_d_ = 8 V. Compared with the erase speed shown in Figure 8b, only 1.7 μs is required for a 2-V *V*_t_ shift. It is reasonable that the erase speed is approximately ten times faster than the program speed because this memory is programmed by BBHH and erased by CHE. Even at only 6-V operation, the P/E speed can be as fast as 120:5.2 μs with a 2-V *V*_t_ shift. The fast P/E speed at such low operation voltage is superior to that demonstrated in previous studies [18-20] and is beneficial to the development of high-performance memory. This favorable result is ascribed to the formation of more trapping sites in the Ti_*x*_Zr_*y*_Si_*z*_O film at 600°C annealing, and hence, more carries can be captured in the traps.

**Figure 9:**
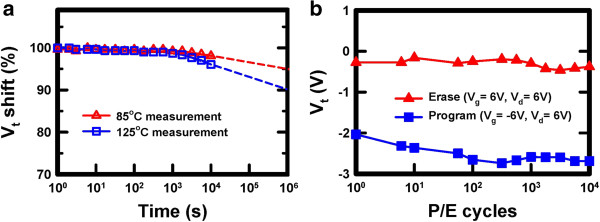
**Reliability characteristics of the Ti**_***x***_**Zr**_***y***_**Si**_***z ***_**O memory. (a)** Retention characteristic of the memory at measurement temperatures of 85°C and 125°C. **(b)** Endurance characteristic of the memory up to 10^4^ program/erase cycles.

A highly reliable charge retention characteristic of the memory is demonstrated in Figure 9a. The normalized *V*_t_ shift is defined as the ratio of the *V*_t_ shift at the time of interest and at the beginning. The curve is obtained under the program conditions of *V*_g_ = −7 V and *V*_d_ = 7 V for 1 ms at 85°C and 125°C, respectively. As time extrapolated up to 10^6^ s, the data retention measured at 85°C shows only 5% charge loss and that at 125°C shows only 10% charge loss. Figure 9b shows the endurance characteristics of the Ti_*x*_Zr_*y*_Si_*z*_O memory. The measurement conditions are *V*_g_ = −6 V and *V*_d_ = 6 V for programming and *V*_g_ = *V*_d_ = 6 V for erasing. Despite a small drift of the threshold voltage for both P/E operations, the memory window remained at around 2 V after 10^4^ P/E cycles. No substantial window narrowing was observed. The threshold voltage downward shift is mainly caused by the interface trap generation and hole trapping in the tunneling oxide.

The electrical performance of the Ti_*x*_Zr_*y*_Si_*z*_O memory is summarized in Table 1 and compared with other sol–gel-derived memories [8,13,21]. As seen in the table, the Ti_*x*_Zr_*y*_Si_*z*_O memory in this study exhibits improved electrical performance, particularly in retention properties. The Ti_*x*_Zr_*y*_Si_*z*_O memory at either 600°C or 900°C annealing can be operated at much higher erase speeds compared to other materials. This is because the erase of the Ti_*x*_Zr_*y*_Si_*z*_O memory is operated by CHE. Moreover, the operation voltage of the sol–gel-derived Ti_*x*_Zr_*y*_Si_*z*_O memory can be decreased to only 6 V, without sacrificing its performance.

**Table 1:** **Comparison of P/E speed and data retention of the sol–gel-derived high-*κ *memory devices**

	**This work (Ti**_***x***_**Zr**_***y***_**Si**_***z***_**O with 600°C annealing)**	**Ti**_***x***_**Zr**_***y***_**Si**_***z***_**O NC with 900°C annealing**[13]	**Zr**_***x***_**Hf**_***y***_**Si**_***z***_**O NC with 900°C annealing**[6]	**HfSi**_***x***_**O**_***y***_**with 900°C annealing**[21]
Program speed (2-V shift)	1.6 × 10^−5^ s	2.4 × 10^−5^ s	3 × 10^−5^ s	2 × 10^−2^ s
	(*V*_g_ = −8 V, *V*_d_ = 8 V)			
	1.2 × 10^−4^	(*V*_g_ = −8 V, *V*_d_ = 8 V)	(*V*_g_ = 10 V, *V*_d_ = 9 V)	(*V*_g_ = *V*_d_ = 10 V)
	(*V*_g_ = −6 V, *V*_d_ = 6 V)			
Erase speed (2-V shift)	1.7 × 10^−6^ s	1.9 × 10^−6^ s	2 × 10^−3^ s	5 × 10^−5^ s
	(*V*_g_ = *V*_d_ = 8 V)			
	5.2 × 10^−6^ s	(*V*_g_ = *V*_d_ = 8 V)	(*V*_g_ = −10 V, *V*_d_ = 9 V)	(*V*_g_ = ^−^10 V, *V*_d_ = 10 V)
	(*V*_g_ = *V*_d_ = 6 V)			
Retention at 85°C	5% loss	12% loss	11% loss	20% loss
	(10^6^ s)	(10^6^ s)	(10^6^ s)	(only 10^4^ s)
Retention at 125°C	10% loss	22% loss	30% loss	NA
	(10^6^ s)	(10^6^ s)	(10^6^ s)	

*NC* nanocrystal.

## Conclusion

We demonstrated a high-performance sol–gel-derived Ti_*x*_Zr_*y*_Si_*z*_O memory in this study. The memory exhibits a notable hot hole program characteristic, and hence, a much higher erase speed is achieved. The barrier height for the Ti_*x*_Zr_*y*_Si_*z*_O film to silicon oxide was estimated to be approximately 1.15 eV for hole trapping, using the Poole-Frenkel emission model. Unlike other sol–gel-derived memories that require a higher temperature annealing process, this Ti_*x*_Zr_*y*_Si_*z*_O memory with relatively low-temperature annealing exhibits excellent electrical performance such as low-voltage operation, fast P/E speed, and robust data retention.

## Abbreviations

BBHH: Band-band hot hole; CHE: Channel hot electron; J/E: Current density; P/E: Program/erase; SiCl4: Silicon tetrachloride; RTA: Rapid thermal annealing; SONOS: Silicon-oxide-nitride-oxide-silicon; TEM: Transmission electron microscopy; TiCl4: Titanium tetrachloride; ZrCl4: Zirconium tetrachloride; XPS: X-ray photoelectron spectroscopy.

## Competing interests

The authors declare that they have no competing interests.

## Authors' contributions

Y-MC, S-HL, Y-PH, and C-CW carried out the experiment and measurement. J-YW and C-CW prepared the manuscript. W-LY and C-CW technically supported the study. All authors read and approved the final manuscript.
